# Supplier-induced demand for psychiatric admissions in Northern New England

**DOI:** 10.1186/1471-244X-11-146

**Published:** 2011-09-09

**Authors:** Bradley V Watts, Brian Shiner, Gunnar Klauss, William B Weeks

**Affiliations:** 1 Department of Psychiatry, Dartmouth Medical School, VA Medical Center, 215 North Main Street, White River Junction, VT 05009, USA; 2 Department of Anesthesiology, Wake Forest University School of Medicine, 100 Medical Center Boulevard, Winston-Salem, NC 27157, USA; 3 The Dartmouth Institute for Health Policy and Clinical Practice, 46 Centerra Parkway, Box 203, Lebanon NH 03766, USA

## Abstract

**Background:**

The development of hospital service areas (HSAs) using small area analysis has been useful in examining variation in medical and surgical care; however, the techniques of small area analysis are underdeveloped in understanding psychiatric admission rates. We sought to develop these techniques in order to understand the relationship between psychiatric bed supply and admission rates in Northern New England. Our primary hypotheses were that there would be substantial variation in psychiatric admission across geographic settings and that bed availability would be positively correlated with admission rates, reflecting a supplier-induced demand phenomenon. Our secondary hypothesis was that the construction of psychiatric HSAs (PHSAs) would yield more meaningful results than the use of existing general medical hospital service areas.

**Methods:**

To address our hypotheses, we followed a four-step analytic process: 1) we used small area analytic techniques to define our PHSAs, 2) we calculated the localization index for PHSAs and compared that to the localization index for general medical HSAs, 3) we used the number of psychiatric hospital beds, the number of psychiatric admissions, and census data to calculate population-based bed-supply and psychiatric admission rates for each PHSA, and 4) we correlated population-based admission rates to population-based psychiatric bed supply.

**Results:**

The admission rate for psychiatric diagnosis varied considerably among the PHSAs, with rates varying from 2.4 per 100,000 in Portsmouth, NH to 13.4 per 100,000 in Augusta, ME. There was a positive correlation of 0.71 between a PHSA's supply of beds and admission rate. Using our PSHAs produced a substantially higher localization index than using general medical hospital services areas (0.69 vs. 0.23), meaning that our model correctly predicted geographic utilization at three times the rate of the existing model.

**Conclusions:**

The positive correlation between admission and bed supply suggests that psychiatric bed availability may partially explain the variation in admission rates. Development of PHSAs, rather than relying on the use of established general medical HSAs, improves the relevance and accuracy of small area analysis in understanding mental health services utilization.

## Background

Small area analysis is a health services research technique that facilitates geographic comparison of health services utilization rates [1]. Using this technique, researchers have consistently documented the existence of supplier-induced demand [2] for health services [3-5]. Rates of procedures--such as tonsillectomy, prostatectomy, and hysterectomy [6]--and inpatient hospitalization rates for general medical illnesses--such as back problems, gastroenteritis, and heart failure [7]--have been shown to be related more strongly to the supply of a service than to the need for that service [8]. While small area analysis has not helped health systems determine the optimal supply of health services, it is clear that small areas with the highest utilization rates experience the worst health outcomes even in the face of similar disease burdens [9,10]. This has spurred concerns that an oversupply of health care may worsen health outcomes for a population [10]. Chief among these concerns is that once the true need for a health service has been served, market forces dictate that excess supply must be consumed by those who do not actually need the services and are therefore exposed to the risk without the potential for benefit [2]. While some conditions always merit treatment, others--so-called high-variation conditions--tend to be treated more intensively in the presence of excess resources [7]. The development of the Dartmouth Atlas of Healthcare has facilitated the application of small area analysis to national datasets and allowed the identification of this phenomenon on a local level,[11] as in reports of the over-provision of cardiac surgery in Reading, California [12,13] and of unusually high rates of coronary stent procedures in Elyria, Ohio [14,15]. Recent literature has been more critical of the concept of supply inducted demand in medicine. While there is general agreement that utilization and supply are correlated, there is less agreement regarding the meaning and drivers of this association [16].

While small area analysis has been extensively applied to hospital-based medical and surgical services, there has been little application to hospital-based psychiatric services. A 1995 analysis of psychiatric inpatient admission patterns in Iowa found higher rates in small areas with more primary care physicians, psychiatrists, and inpatient psychiatric units [17]. The authors concluded both that the differences were unlikely to be related to differences in need and that demand for inpatient psychiatry services was, in fact, sensitive to supply. However, there were several limitations to this analysis. First, the authors used standard hospital service areas (HSAs), which are based upon where most Medicare recipients who live in contiguous zip codes obtain general inpatient hospital services. As there are many more general hospitals than there are psychiatric hospitals and general hospitals with psychiatric units, most of the HSAs did not contain a psychiatric unit. This method did not allow researchers to account for use of inpatient psychiatric services in neighboring HSAs. An earlier analysis grouped these HSAs by county into politically-defined community mental health center (CMHC) catchment areas and found that access to CMHC resources induced demand for inpatient psychiatric admissions [18]. However, the CMHC catchment areas were not necessarily the same or even intended to be the same as catchment areas for inpatient psychiatric units. Perhaps the most comprehensive study of geographic variation in inpatient mental health care examined county variation in New York [19]. This study found that population variables such as poverty and population density were highly correlated with mental health service utilization. Furthermore, even when controlling for these factors, proximity to inpatient care resulted in increased utilization.

Often small area analysis has examined specialty care by aggregating HSAs into larger hospital referral regions (HRRs). HRRs are based upon where most Medicare recipients living in contiguous zip codes obtain heart surgery and neurosurgery [11]. While useful for understanding geographic health service use patterns in expensive, highly technical procedures, these HRRs may not be as useful for understanding utilization of psychiatric inpatient services. As large inpatient psychiatric facilities are sometimes located in areas that do not have a medical referral hospital and as there is no analogous hierarchy of complexity in psychiatric units (for example, university-based psychiatric units do not necessarily offer more complex or technical procedures than community-based psychiatric units as is the case in medical hospitals), it is unlikely that geographic patterns of referral would be the same. A final concern with using standard HSAs is that patients admitted to inpatient psychiatric units tend to be younger than patients admitted to inpatient general medical units. As a result, fewer patients are eligible for Medicare; Medicare billing data may not be the most appropriate information to use to define HSAs in this population. Another major limitation of the Iowa and New York State studies is that they looked at only one state. As HSAs often cross state lines, it may be more meaningful to look at a region rather than a single state.

Another study examined geographic variation in inpatient psychiatric admission in New York City [20]. The authors relied on zip codes as their unit of analysis and did not construct hospital service areas. They found that patients residing in a zip code where an inpatient psychiatric unit was located were more likely to be admitted. However, this analysis is subject to the same fallacy as the Iowa and New York State data, given that many zip codes did not have an inpatient psychiatric unit and that there is no reason to believe patients obtain their medical care within their zip code.

Therefore, if the intent is to study geographic variation in inpatient psychiatric admissions, it would be most helpful if: 1) each HSA--in this case Psychiatric HSAs or PHSAs--had at least one inpatient psychiatric hospital located within its boundaries, 2) we knew the capacity (number of psychiatric beds) of these inpatient psychiatric units rather than simply whether they existed or not, 3) we used the most population-relevant data (including using all adult age groups) to define our PHSAs, 4) we recognized that in some areas, especially along interstate borders, people are likely to live in one state and obtain healthcare in another, and 5) we parted from the assumption that these PHSAs will have some hierarchical regional organization as seen in general medical HSAs.

One of our goals in this study was to define the adaptations that would make small area analysis more relevant to the study of psychiatric care. We chose inpatient psychiatric treatment as a starting point both because of the large cost strain it places on the mental health treatment system and because of the previously-documented possibility of supplier-induced demand in this sector. Inpatient psychiatric treatment is an integral part of mental health treatment in the United States. In 2000, an estimated 215,221 inpatient psychiatric beds in 3,202 hospitals accommodated 2,153,874 psychiatric hospitalizations at a cost of almost 33 billion dollars [21]. The large capacity of and costs associated with inpatient psychiatric care in the US reflects its central role in the provision of care for mental health patients. Overall, 74% of all mental health care dollars are spent on inpatient care: Medicare spends 83% of its mental health care budget on inpatient treatment,[22] 65% of State mental health spending is for inpatient care, and Blue Cross/Blue Shield recently reported that 66% of their mental health care spending was for inpatient care [23]. Despite the volume and cost of inpatient psychiatric care, there is very little research regarding its effectiveness. Indeed, no randomized clinical trials have demonstrated effectiveness for inpatient care in a general psychiatric population [24].

We had three hypotheses in conducting this study, related to the actual subject at hand (inpatient psychiatric admissions) and the method to be used (applying small area analysis to mental health services). Our primary hypotheses were that there would be substantial variation in psychiatric admission across geographic settings and that bed availability would be positively correlated with admission rates, reflecting a supplier-induced demand phenomenon. Our secondary hypothesis was that the construction of psychiatric HSAs would yield more meaningful results than the use of existing general medical HSAs. This article reports the first small area analytic study of mental health services utilization using discipline-specific techniques. Our approach does not discount the considerable previous literature which has demonstrated other important drivers of inpatient mental health care including population factors (poverty and prevalence of mental illness), ambulatory treatment resources (availability and quality), and geographic proximity of inpatient service. Our focus on the quantity of inpatient mental health beds reflects the relative paucity of research regarding this variable compared to the important variables above.

## Methods

To address our hypotheses, we followed a four-step analytic process: 1) we used small area analytic techniques to define our PHSAs, 2) we calculated the localization index for psychiatric hospital service areas and compared that to the localization index for general medical HSAs, 3) we used the number of psychiatric hospital beds, the number of psychiatric admissions, and census data to calculate population-based bed-supply and psychiatric admission rates for each psychiatric service area, and 4) we correlated population-based admission rates to population-based psychiatric bed supply. This study was approved by Dartmouth Medical School's Committee for the Protection of Human Subjects, Hanover, NH (CPHS # 20009).

### Determination of psychiatric hospital service areas

We obtained 1997 hospitalization datasets for the states of Maine, New Hampshire, and Vermont from the Maine Health Care Finance Commission (Augusta), the New Hampshire Department of Health and Human Services (Concord), and the Vermont Department of Health (Burlington), respectively. We chose 1997 both because it allowed us to use 2000 census data and because it allowed comparison with the Dartmouth Atlas of Health Care general medical HSAs, which had last been calculated for the 1996 edition [13]. Each dataset included the patients' age, gender, home ZIP Code, discharge diagnosis, length of stay, and the ZIP Code of the treating hospital. Only hospitalizations related to those Diagnostic Related Groups (DRG) specific to psychiatry were used for this project. These included DRG 424-437 corresponding to diagnosis of Depression, Psychosis, Anxiety, Alcohol Dependence, Drug Dependence, and Organic Mental Condition. We excluded all hospitalizations of patients under age 18.

We defined PHSAs for these three states using the standard methods of small area analysis [6]. First, we used the home ZIP codes of patients admitted with psychiatric disorders to create a patient origin matrix for all adult psychiatric admissions. We excluded patients whose home ZIP Code was not located in Maine, Vermont, or New Hampshire. Next, we determined which hospitals provided care to patients in each ZIP Code. For each patient ZIP code we identified the hospital which had a plurality of all psychiatric admissions. Finally, neighboring ZIP Codes were assigned to PHSAs to form contiguous ZIP Code defined areas. PHSAs were allowed to have as many or as few component ZIP Codes as dictated by the data so long as they remained contiguous. A PHSA could contain more than one hospital under two circumstances: when two or more hospitals were located within the same zip code or when combining hospitals allowed for a more contiguous geographic area.

### Determination of the Localization Index and comparison with the Dartmouth Atlas of Health Care

We used standard techniques to determine the extent which patients received care within the PHSA, known as the "localization index."[13] The localization index is the percentage of psychiatric admissions in a given PHSA that are admitted to a hospital within the same PHSA. We determined the localization index for each PHSA. In addition, mean total localization index for the PHSA model was determined by combining the results of localization indexes for each PHSA. We then compared the PHSA with the general medical HSAs developed using all Medicare discharges for the *Dartmouth Atlas of Health Care*. Using the previously defined general medical HSAs, we determined a localization index using the total psychiatric admission data obtained from the states. We also determined an overall localization index for the general medical HSAs.

### Calculation of the population-based psychiatric hospital bed supply and psychiatric admission rate

PHSAs were defined by both the number of hospitals that provided psychiatric admissions (range 1-4 hospitals per PHSA) and by a geographically-defined group of ZIP Codes. To determine the supply of psychiatric beds available, we simply added the number of psychiatric beds associated with all of the hospitals in each PHSA. The psychiatric bed numbers were determined by obtaining the number of licensed psychiatric beds in 1997 from each state department of health. We confirmed this bed number by discussion with staff at each hospital. Similarly, we determined the total number of admissions associated with each PHSA using the admission dataset. Because total population of each PHSA varied, we obtained population counts from the 2000 census. As we were only interested in adult psychiatric care we only used census populations 18 years and older. We used these data to calculate the number of beds and admissions per 10,000 persons in the associated geographic area.

### Analysis

To give readers a sense of the inherent differences in psychiatric admissions at the State level, we provide an analysis of the demographics and primary diagnosis of psychiatric admissions in 1997. We used ANOVA to compare continuous variables and the chi-square test to compare categorical variables. We used the Spearman's correlation statistic to compare the population based numbers of psychiatric beds and admission rates because the bed supply was not normally distributed [25]. All statistical analyses were conducted using STATA 8.0 (College Station, TX).

## Results

In 1997, there were 22,503 total admissions for adults with primary psychiatric illnesses in the three States examined (Table 1). Female patients accounted for 53% of admissions. The average length of stay was nine days with a median length of stay of six days. The most common diagnostic groups admitted were psychosis-including major depression, schizophrenia, and bipolar disorder (47%), substance use disorder (23%), and neurosis-including axis II disorders (12%). Patient age groups were 18-24 years (14%), 25-44 years (48%), 45-64 years (22%), 65-74 years (7%), and > 75 years (11%). At the State level, there was no significant variation in the average length of stay, gender, or ages of admission. With the exception of different rates of admission for substance disorders, diagnoses were similarly distributed across states.

**Table 1:** Characteristics of psychiatric admissions by state

	All States	NH	ME	VT
Total Population	3,119,527	1,235,791	1,274,915	608,821
Psychiatric Admissions	22,503	6,842	12,319	3,342
Percent Female	53%	56%	51%	57%
Length of Stay (days)				
Mean (SD)	8.97 (11.83)	8.22 (10.12)	9.6 (13.81)	8.17 (9.42)
Median	6	6	7	6
Range	1-297	1-297	1-252	1-128
Diagnosis				
Psychosis	47%	47%	45%	55%
Substance Use Disorder	23%	22%	28%	11%
Neurosis	12%	11%	10%	16%
Detoxification	5%	4%	5%	2%
Organic Disorder	5%	7%	4%	5%
Adjustment Disorder	4%	3%	3%	6%
Personality Disorder	2%	3%	1%	4%
Other	3%	1%	4%	1%
Age				
18-24	14%	12%	15%	13%
25-44	48%	47%	48%	49%
45-64	22%	22%	20%	22%
65-74	7%	7%	7%	6%
> 75	11%	12%	10%	10%

*Totals may be > 100% due to rounding

**p < 0.05

Table 2 shows the admission rate in each PHSA. Of the twenty-five PHSA, fifteen included only one hospital with psychiatric beds, six had two hospitals with psychiatric beds, three had three hospitals with psychiatric beds, and one had four hospitals with psychiatric beds. The lowest rate was 2.4 admissions per 10,000 populations in Portsmouth, New Hampshire and the highest was 13.4 in Augusta, Maine. The calculated localization index was 0.69, meaning that 69% of the patients admitted from a PHSA went to a hospital within that PHSA. The localization index for psychiatric admissions using the general medical HSAs developed for the *Dartmouth Atlas of Health Care *was 0.23, meaning that general medical HSAs correctly predicted the location of psychiatric care only 23% of the time.

**Table 2:** Psychiatric Admission Rates in Northern New England

PHSA*	Admission Rate/10,000
Portsmouth, NH	2.4
Rochester, NH	3.2
Burlington, VT	3.2
Rutland, VT	4.6
Nashua, NH	5.0
Laconia, NH	5.2
Fort Kent, ME	5.4
Manchester, NH	5.7
Lebanon, NH	5.9
Barre, VT	6.1
Berlin, NH	6.3
Presque Isle, ME	6.3
Bennington, VT	6.5
Concord, NH	6.6
Brattleboro, VT	7.0
Bath, ME	7.1
Rockport, ME	7.3
Bangor, ME	7.4
Keane, NH	8.1
Portland, ME	8.2
Waterville, ME	8.9
Lewiston, ME	9.3
Claremont, NH	9.5
South Portland, ME	10.7
Augusta, ME	13.4

*PHSA: Psychiatric Hospital Service Area

**Figure 1:**
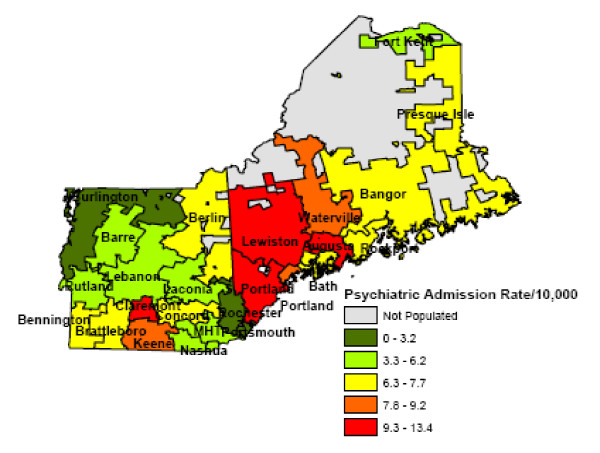
**Psychiatric Hospital Service Areas in Northern New England**.

**Figure 2:**
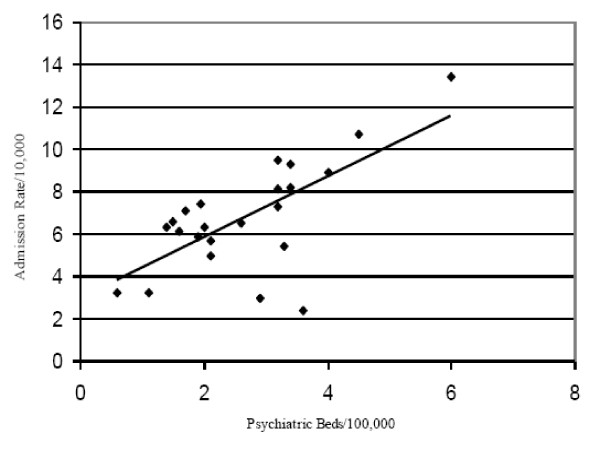
**Population-Based Psychiatric Bed Supply and Admission Rates**. Each dot represents a PHSA; r = 0.71, p = 0.0085.

Figure 1 shows the various psychiatric hospital service areas for Northern New England; PHSAs borders frequently crossed State borders. The map shows the variation in admission rates. In some cases, PHSAs with the very highest psychiatric admission rates are geographically juxtaposed with PHSAs with the very lowest psychiatric admission rates. We found a positive correlation of 0.71 between the supply of beds and the admission rate in a PHSA (p < .001) (Figure 2).

## Discussion

We found that the principles of small area analysis can be applied to inpatient psychiatric care, that use of psychiatric-specific hospitalization to define inpatient psychiatric service areas results in better localization indices than does use of medical and surgical defined hospital service areas, and that the substantial variation in admission rates--which is not fully captured at the individual State level--is associated with increased psychiatric bed supply. The high degree of variability and the association between psychiatric bed supply and psychiatric admission rates suggests a supplier induced demand phenomenon.

Our study represents an initial step in better understanding quality, consistency, and effectiveness of inpatient psychiatric admission across larger geographic settings. Further, our study justifies use of methods specific to psychiatry to analyze mental health admissions. Similar modifications have been used for other services, such as outpatient primary care [26] and outpatient psychiatric services,[27] where established utilization patterns for general inpatient medical care may simply not be relevant. Our findings suggest that, for the purpose of examining psychiatric admissions, the development of psychiatric specific hospital service areas is warranted. Given the large improvement in localization index when comparing our PHSAs to general medical HSAs, we conclude that our findings are more accurate than those found elsewhere.

Our study has several limitations. First, we did not incorporate measures of quality or outcomes into the analysis: such work was beyond the scope of this exploratory study. While the addition of quality and outcomes metrics would not change the variation seen in admission rates, they could substantially change the interpretation of those data. Incorporation of such data would be important in trying to determine an appropriate psychiatric admission rate. Second, we were not able to incorporate different measures of population-based need into the analysis. Should the population of Augusta, ME have substantially higher inpatient psychiatric service needs than the population of Portsmouth, NH, the differences in population based admission rates that we found might be justified. Regardless, the relationship between bed supply and admission rates warrants further investigation. Third, we were not able to correct for alternative methods of treatment provision. It may be that greater levels of intensive outpatient services might have been provided as local substitutes for bed supply, and that such services resulted in lower admission rates. If this is the case, and intensive outpatient services are less costly than inpatient services, highly bedded psychiatric hospital service areas might achieve efficiencies by substituting intensive outpatient treatment for inpatient services. We think that this is unlikely given Hendryx et al.'s finding that access to these services may actually induced added demand for inpatient psychiatric admission [18]. Finally, our analysis was limited to the admission data from the hospitals that provide admission data to the three states examined. State psychiatric hospitals, veterans' hospitals, and hospitals outside the three states that might serve some of each state's population do not provide data to these three states. It is possible that differing patterns of utilization of those inpatients beds was present in different PHSA. These important methodologic limitations similarly limit our primary assertion, that supply inducted demand existed in mental health care in Northern New England. Supplier inducted demand reflects treatment in excess of the treatment provided to improve outcomes and meet patient preferences. As indicated above we have no information regarding patients' outcomes or preferences, thus our main finding must be considered quite preliminary.

## Conclusions

Inpatient psychiatric care remains a central feature of virtually all mental health systems and represents a substantial proportion of overall mental health care costs. The demonstration of large amounts of variability in the rates of inpatient admission begs the question, "What is the appropriate rate?" While we did show an association between inpatient mental health utilization and bed supply, it remains unclear if this relationship reflects too many or too few beds. Furthermore, examination of a singular association such as the one we found in isolation should be done with caution, as previous studies have shown that important population and outpatient mental health characteristics also exert influence on inpatient utilization. Our findings suggest that further research examining geographic variation in the provision of psychiatric services and the relationship of that variation to the supply of services is warranted. We believe that the examination of regions empirically derived from mental health data offers clear advantages over examination of artificially derived boundaries such as states or counties. Our work also suggests that attempts to quantify mental health services (such as examining the number of mental health beds in a region) can add to our understanding. Considerable future work is warranted in this important topic. Expanding the boundaries of study through examination of longitudinal trends would be a significant advancement. In addition, examination of larger geographic regions would aid with fuller understanding of utilization. Considerable prior work has examined population factors that may influence treatment need and treatment utilization. Less work has been accomplished in understanding treatment and provider characteristics that may influence utilization. For example, poorly designed and organized outpatient services could decrease utilization and increase inpatient need, while highly quality outpatient care could decrease inpatient use. Sufficient data granularity is needed in this area. Similarly, we know little about the possible mechanisms through which "supply-inducted demand" occurs (if it even exists). Greater understanding of those mechanisms could add validity regarding the existence of the supplier-induced demand phenomenon. Ultimately, care that results in improved outcomes for patients is valuable, thus understanding the underlying relationship between a treatment type and outcomes is of considerable interest. Establishing relationships between good behavioral health in a population and particular rates of admission would have important implications for determining psychiatric bed availability in a population.

## List of abbreviations

HSA: hospital service area; CMHC: community mental health center; HRR: hospital referral region; PHSA: psychiatric hospital service area; CPHS: committee for the protection of human subjects; ZIP: zoning improvement plan; CPT: current procedural technology; ANOVA: analysis of variance.

## Competing interests

The authors declare that they have no competing interests.

## Authors' contributions

BVW designed the study, obtained the data, and drafted the initial manuscript. BS assisted in data collection and interpretation of the results, and revised the manuscript. GK performed statistical analysis. WBW conceived the study and provided intellectual supervision. All authors read and approved the final manuscript.

## Pre-publication history

The pre-publication history for this paper can be accessed here:

http://www.biomedcentral.com/1471-244X/11/146/prepub
